# Single cell force profiling of human myofibroblasts reveals a biophysical spectrum of cell states

**DOI:** 10.1242/bio.049809

**Published:** 2020-03-24

**Authors:** Thomas B. Layton, Lynn Williams, Huw Colin-York, Fiona E. McCann, Marisa Cabrita, Marc Feldmann, Cameron Brown, Weilin Xie, Marco Fritzsche, Dominic Furniss, Jagdeep Nanchahal

**Affiliations:** 1 The Kennedy Institute of Rheumatology, Nuffield Department of Orthopaedics, Rheumatology and Musculoskeletal Sciences, University of Oxford, Oxford OX3 7FY, UK; 2 MRC Human Immunology Unit, Weatherall Institute of Molecular Medicine, University of Oxford, Headley Way, Oxford OX3 9DS, UK; 3 Botnar Research Centre, Nuffield Department of Orthopaedics, Rheumatology and Musculoskeletal Sciences, University of Oxford, Oxford OX3 7LD, UK; 4 Department of Inflammation Research, Celgene Corporation, San Diego, CA 92121, USA

**Keywords:** Focal adhesion, Myofibroblast, Single cell, Traction force

## Abstract

Mechanical force is a fundamental regulator of cell phenotype. Myofibroblasts are central mediators of fibrosis, a major unmet clinical need characterised by the deposition of excessive matrix proteins. Traction forces of myofibroblasts play a key role in remodelling the matrix and modulate the activities of embedded stromal cells. Here, we employ a combination of unsupervised computational analysis, cytoskeletal profiling and single cell traction force microscopy as a functional readout to uncover how the complex spatiotemporal dynamics and mechanics of living human myofibroblast shape sub-cellular profiling of traction forces in fibrosis. We resolve distinct biophysical communities of myofibroblasts, and our results provide a new paradigm for studying functional heterogeneity in human stromal cells.

## Abstract (precis)

**Summary:** High resolution traction force microscopy at single-cell level reveals the biophysical landscape of primary human myofibroblasts.

## INTRODUCTION

Analysis of single-cell biology has illuminated crucial biological processes in health and multiple diseases (Giustacchini et al., 2017; Kinchen et al., 2018). These techniques quantify surrogate markers, such mRNA molecules or protein abundance and subsequently ascribe functions to discrete cell types (Villani et al., 2017; Aizarani et al., 2019). However, pertinent to all fibrotic diseases, the functional characterisation of primary human stromal cells remains incomplete.

Myofibroblasts are crucial mediators of normal wound healing and contribute excessive extracellular matrix (ECM) during fibrosis (van Beuge et al., 2016; Wynn and Ramalingam, 2012). A central function of myofibroblasts is the generation of traction force, which plays a key role in remodelling the matrix and also modulates the activities of the embedded stromal cells (Liu et al., 2015; Hinz and Gabbiani, 2003; Goffin et al., 2006). Despite several studies quantifying fibroblast traction force (Marinković et al., 2012; Rayan et al., 1996), we still lack an understanding of this process in human myofibroblasts (Wang et al., 2003). Indeed, we are yet to obtain precise force measurements in myofibroblasts that would enhance our understanding of their function (Hinz et al., 2001). At present, we are limited to force measurements of groups of cells without a single-cell perspective. In addition, we do not know whether distinct myofibroblast populations exist based on their biophysical characteristics. Single-cell force measurements would address these questions and allow the determination of functional heterogeneity in primary human myofibroblasts.

Although techniques such as culture force monitoring and collagen populated lattice modelling have elucidated critical regulatory processes of stromal cell contraction (Tseng et al., 2011; Polacheck and Chen, 2016), they report measurements of representative groups of cells and lack the required resolution to interrogate single-cell force profiles. Several techniques have been developed to measure force generation in individual cells and one that has gained prominence is traction force microscopy (TFM) (Colin-York et al., 2019a,b, 2016, 2017). TFM is based on the reconstruction of forces from a measured displacement field and uses hydrogels of defined stiffness with fiducial markers to track substrate deformations (Colin-York et al., 2017). Importantly, TFM allows for traction forces to be quantified at sub-cellular resolution (∼1–2 µm), enabling distinct biophysical structures within cells to be studied. Hence, we hypothesised that TFM could be used to characterise the biophysical properties of human myofibroblasts.

Dupuytren's disease (DD) is a localised fibrotic condition of the hand and commonly treated by excision of the diseased fibrotic cords and associated myofibroblast-rich nodules (Verjee et al., 2009, 2010, 2013). Therefore, DD provides an ideal model disease to study human myofibroblasts. Here, we first defined the *in vivo* tissue mechanics in fibrosis and subsequently probed primary human myofibroblasts in a comparable mechanical environment. We uncovered distinct biophysical profiles of myofibroblast force generation and diverse cytoskeletal topologies which were modulated during cell spreading. Crucially, we provide the first evidence for distinct myofibroblast subpopulations with unique force profiles and characterise the mechanics of myofibroblast collagen interaction at subcellular resolution, which has important implications for understanding the functional roles of myofibroblasts in human fibrosis.

## RESULTS AND DISCUSSION

### Mapping the mechanical landscape in human fibrosis

**Fig. 1.**
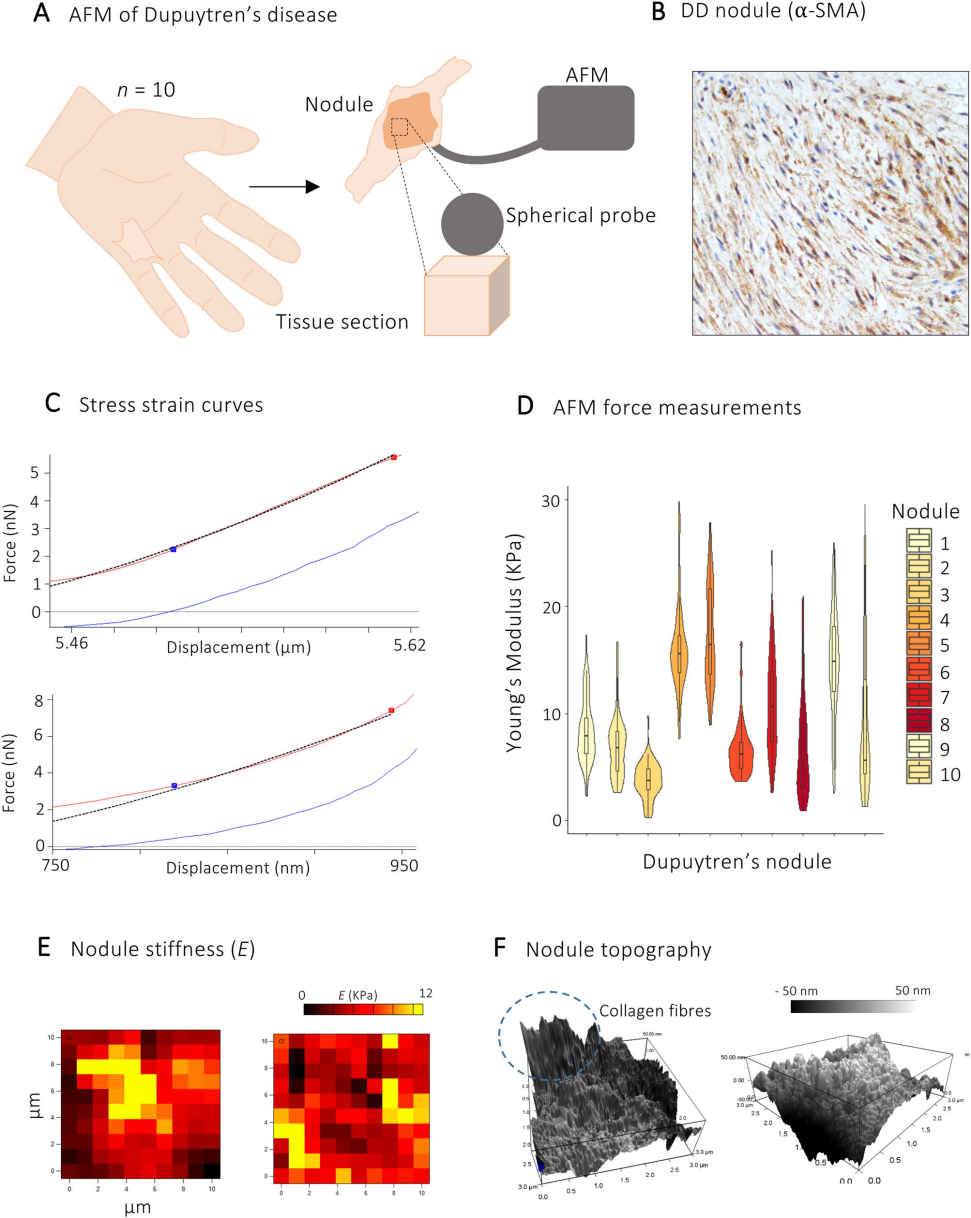
**Mapping the mechanical landscape in human fibrosis.** (A) Schematic demonstrating experimental workflow for measurements of tissue stiffness. Central nodular tissue was dissected to cube-like structures and these were then sectioned in 30 μm slices. Tissue slices were probed using micro-indentation with a 5 μm sphere-tip probe mounted on the AFM cantilever. (B) Immunohistochemistry slide of DD nodule showing α-SMA staining. (C) Stress strain curves of AFM protocols for the application of mechanical force and measurements of two separate Dupuytren's nodules. (D) Violin plot showing Young's modulus of ten Dupuytren's nodules. Each point represents one micro-indentation measurement (*n*=300 per nodule). (E) Exemplar mechanical maps obtained by atomic force microscopy. Each map was derived from an independent DD patient nodule and provides a 100-point profile of Young's modulus (colour bar is inset). (F) Surface profiles showing topography of Dupuytren's nodules with linear structures of collagen fibres. Bounding box heights scaled at 100 nm.

The mechanical environment directly modulates stromal cell function and force measurements. Therefore, we first sought to define the native tissue mechanics of the myofibroblast-rich DD nodules. To quantify the Young's modulus (*E*) of nodules, we performed nano-indentation using atomic force microscopy (AFM) (Fig. 1A–C). The AFM cantilever was equipped with a 5 µm sphere-tipped probe, allowing measurements to be taken at cell-relevant scales. We observed a large range of nano-indentation measurements across individual nodules (Fig. 1D), suggesting that the structure was relatively heterogeneous. The nodules had a Young's modulus of 9 kPa (±5 kPa SEM) (Fig. 1D,E), comparable to values reported for other fibrotic tissues that range from ∼10­–20 kPa (Liu and Tschumperlin, 2011; Karki and Birukova, 2018; Liu et al., 2016, 2015).

Next, we explored the topography of DD nodules using high resolution AFM surface scans. In accordance with the stiffness measurements, the structure of the DD nodule was heterogeneous, with readily identifiable collagen bundles in a linear arrangement (Fig. 1F). The collagen bundles were present in an organised pattern that correlates with previous histological descriptions of nodules (Badalamente et al., 1983).

### Characterising the biophysical profile of myofibroblast force foci

**Fig. 2.**
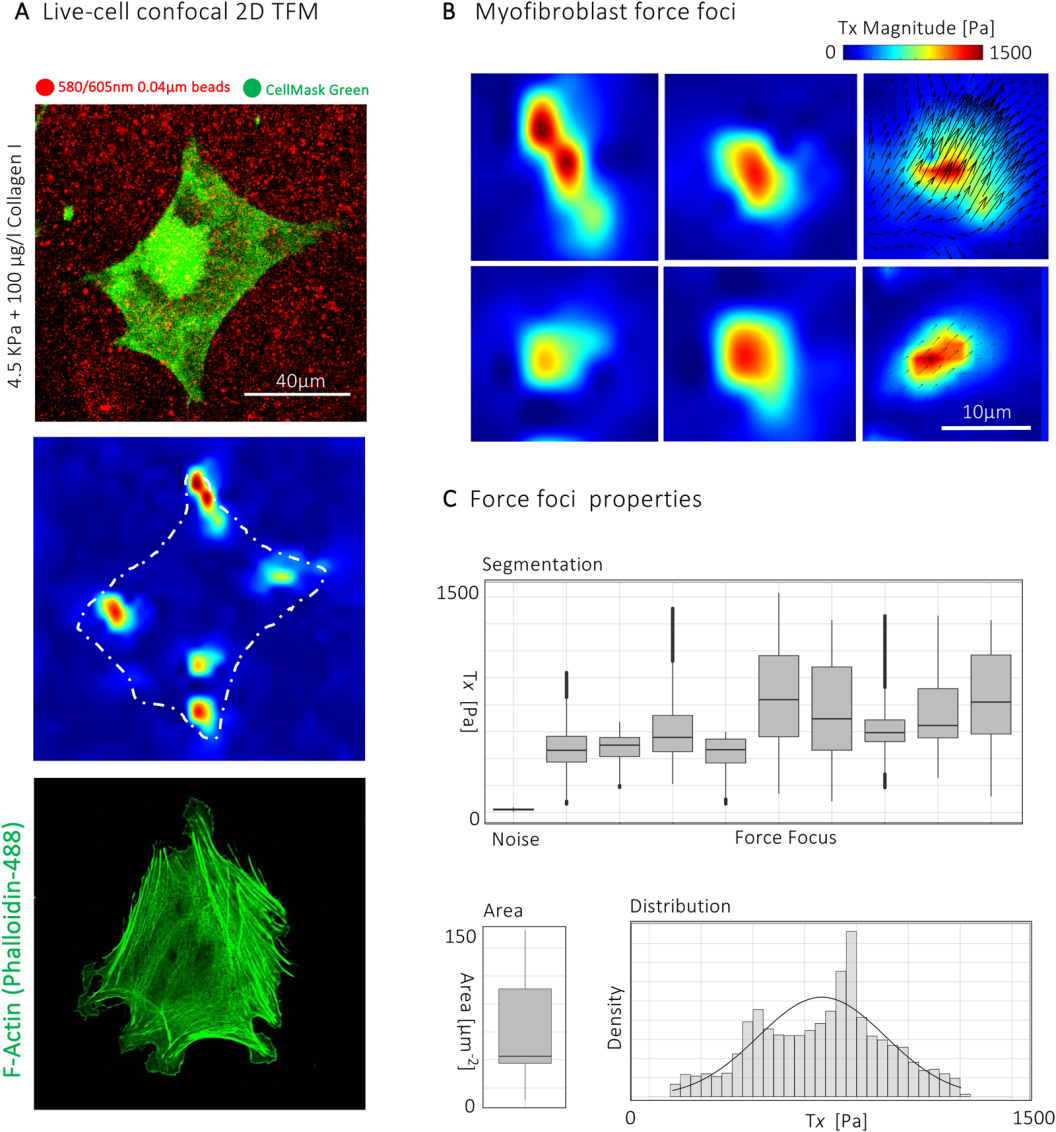
**Characterising the biophysical profile of myofibroblast force foci.** (A) Top two panels, confocal image of Calcein AM tagged myofibroblast (green) on 4.5 kPa PAA gel with marker beads (red) and corresponding traction stress heatmap showing localised areas of high traction force in red. Bottom panel, confocal image of immunofluorescence staining of F-actin of myofibroblast on 4.5 KPa hydrogel. (B) Traction stress plots showing localised areas of high traction force. Images represent single force foci from two myofibroblasts measured using traction force microscopy. Arrows signify vector fields of bead displacement used to track cellular forces. (C) Properties of force foci in human myofibroblasts. Box and whisker plots showing the area and range of forces in segmented force foci in human myofibroblasts with histogram showing the distribution of forces. *n*=9 force foci from three independent experiments.

After defining the native tissue stiffness of DD nodules, we performed traction force microscopy on gels with a Young's modulus ranging from 0.5 kPa to 20 kPa. Cells were seeded on gels coated with type I collagen as this represents the most abundant ECM protein in nodules. We found that cells seeded on hydrogels with a Young's modulus of 4.5 kPa allowed reproducible quantification of bead displacements whilst lying within the range of stiffness found on AFM measurement of the nodules. (Fig. S1A–E). TFM revealed that the average cellular force was 125 Pa (range 21–349 Pa) (Fig. S2A,B), similar to previous reports of stromal cells (Munevar et al., 2001; Morin et al., 2014). The range of force generation by myofibroblasts suggests great heterogeneity in this population, with some cells being highly contractile and others exerting far weaker force. Peak traction force was 634 Pa (range 93–2096 Pa), also showing a wide range with some cells able to produce a force of several kPa (Fig. S2A,B). Fully spread myofibroblasts were characterised by a distinctive distribution of cellular force (Fig. 2A,B), with oval, discrete force foci of high force magnitude, with the remaining cell area exerting a low force that was comparable to background noise levels in the gel (Fig. 2C; Fig. S3A,B).

Force foci in fully spread myofibroblasts varied in number, with most cells containing three to five. Their overall topography was highly conserved, with localised areas of high force magnitude adjacent to a more smooth distribution of lower force (Fig. 2C; Fig. S3A,B). Individual force foci were characterised by traction forces that followed a Gaussian distribution (Fig. 2C). Segmentation of the force foci confirmed that the remaining cell surface generated little force above background noise levels (Fig. 2C). Therefore, adhesion of myofibroblasts to the matrix is through these force foci and enables the application of traction force during remodelling. Together, these provide the first detailed subcellular measurements of force generation in human myofibroblasts on a substrate that reflects the *in vivo* mechanical environment. Notably, the topography and size of myofibroblast force foci (8.1±3.3 µm) mirrored the size of supermature focal complexes (suFA 8.5±3.6 μm to 9/9±3.1 μm) previously described in myofibroblasts (Goffin et al., 2006) and corresponding fibrillar ECM proteins (Fig. 2B; Fig. S3A,B).

### Traction forces are modulated during myofibroblast spreading

**Fig. 3.**
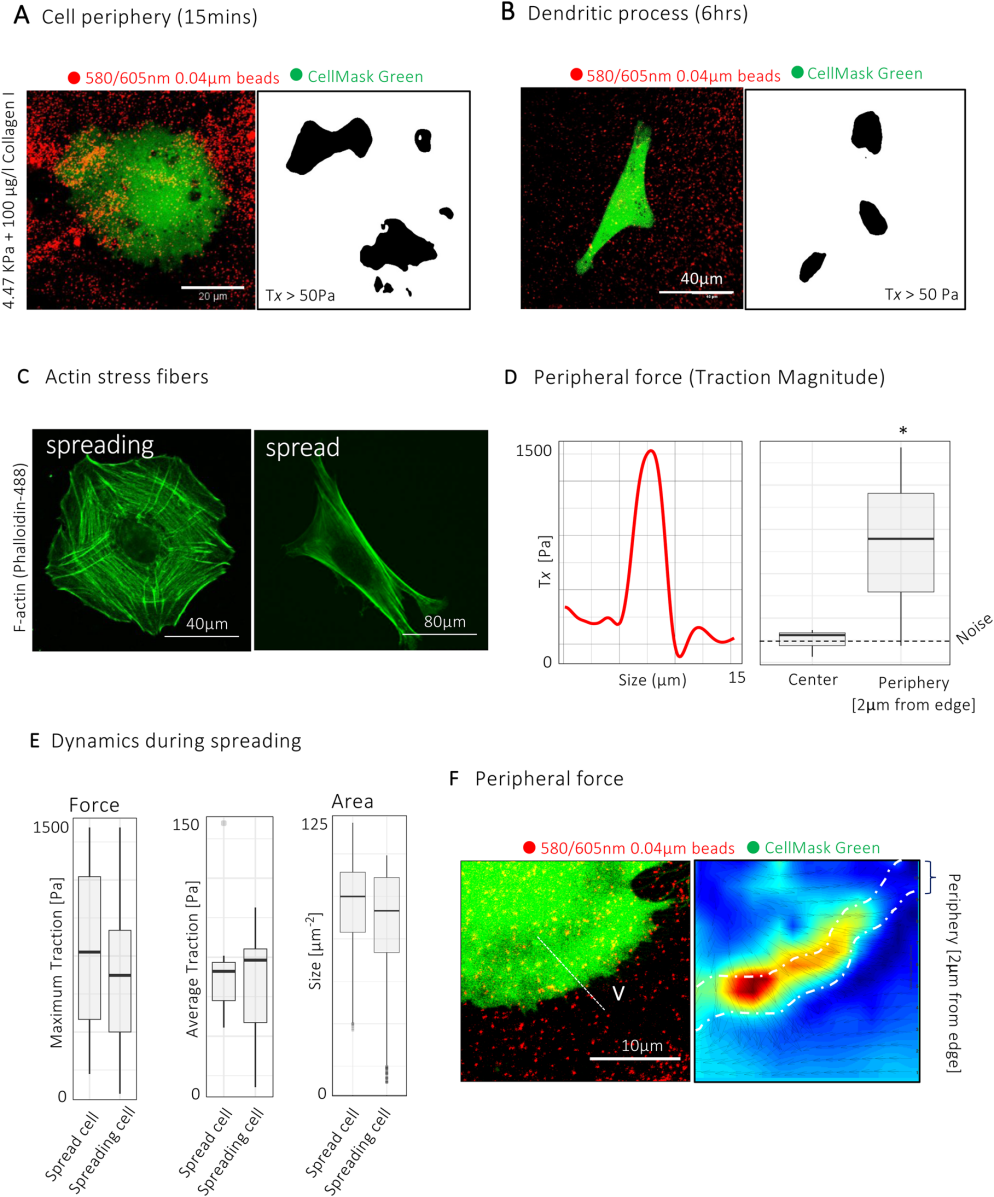
**Traction force** **is** **modulated during myofibroblast spreading.** (A,B) Confocal images of Calcein-AM-tagged myofibroblasts (green) seeded on 4.5 kPa Polyacrylamide (PAA) hydrogel (A) 15 min and (B) 60 min after seeding, with corresponding binary plots of traction forces above noise level. Black regions represent areas of traction force above noise level (∼50 Pa). T_x_=Traction. (C) Confocal image of F-actin staining (Phallodin-488) in spreading (15 min) and fully-spread myofibroblast (>60 min) showing organisation of F-actin and stress-fibre topology. (D) Line profile of traction-force magnitude across line V in F showing the distribution of force along one representative cell periphery and box and whisker plot showing mean traction force at cell periphery (2 μm from cell edge) compared to the remaining cell surface (background) during spreading (15 min after seeding cells). *n*=20 myofibroblasts from three independent donors. *P<0.05 (Wilcoxon Rank Sum test). (E) Box and whisker plots showing maximum and mean traction forces, and area of traction force above noise levels in spreading and spread myofibroblasts. *n*=>12 myofibroblasts from three independent experiments. (F) Confocal image of spreading myofibroblasts (green) and corresponding traction stress heatmap.

Next, we used single-cell force measurements to explore the spatiotemporal dynamics of myofibroblast force during cell spreading (Fig. 3A,B). We observed a distinct evolution of force profiles during spreading over time. On initial contact with the hydrogels, myofibroblasts were circular and traction forces localised to the cell periphery (Fig. 3A). Immunofluorescence staining of spreading myofibroblasts confirmed that these peripheral structures were characterised by dendritic actin networks with small, radially orientated F-actin stress fibres (Fig. 3C). When the cell was fully spread, cellular traction forces shifted to the discrete oval regions described previously (Fig. 3B). Segmentation of cellular forces at the cell periphery (2 µm from cell edge) showed the remaining cell area exerted forces comparable to the background level (Fig. 3D). Comparing the traction force at early and late time points of cell spreading demonstrated that maximum and average traction forces remained constant, as did the area of traction forces above noise levels (Fig. 3E). Collectively, these results provide evidence for distinct biophysical profiles within myofibroblasts undergoing spreading, with maintenance of traction force during this process (Fig. 3E,F).

### Distinct biophysical and cytoskeletal subpopulations in human myofibroblasts

**Fig. 4.**
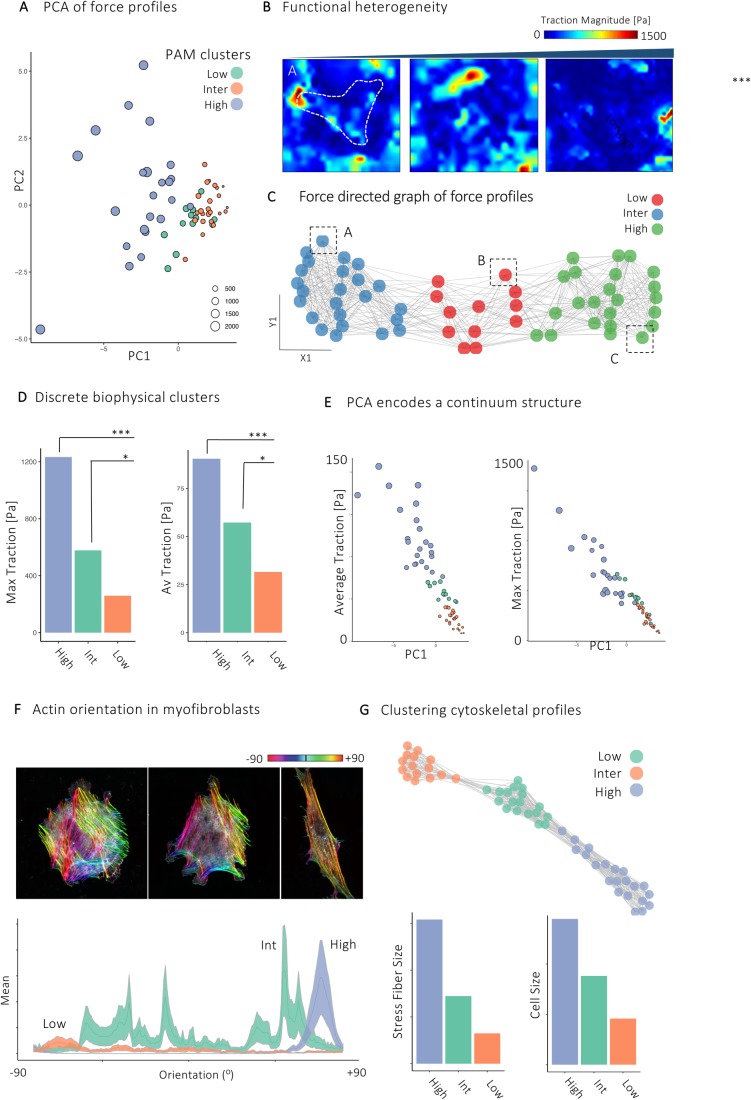
**Distinct biophysical subpopulations of human myofibroblasts.** (A) Scatter plot projecting single-cell force profiles along the first two principal components (PC1 and PC2). Each point represents one cell and cells are coloured by PAM cluster (*n*=59 cells). (B) Corresponding traction force heatmaps of cells identified in dashed black squares in (C). (C) *k*-NN graph of PCA meta-signatures (force profiles) coloured by Louvain cluster. Each point represents a single cell (*n*=59 cells). (D) Bar plots of maximum and mean traction forces per PAM cluster. *P-value<0.05 and ***P-value<0.01 (Wilcoxon Rank Sum test, *n*=59 cells). (E) Scatter plot projecting maximum and average traction force per cell along the first principal component. Each point represents one cell and size represents maximum traction force (*n*=59, from >5 independent donors). (F) Immunofluorescence of actin orientation in representative myofibroblast types with corresponding quantification of actin stress fibre orientation (*n*=59 myofibroblasts from three independent experiments). (G) *k*-NN graph of myofibroblast cytoskeletal and morphological features (cell area, aspect ratio, stress-fibre length and stress-fibre orientation) with corresponding bar plots of stress-fibre length and cell size (*n*=59 myofibroblasts from three independent experiments). Scale bar: 20 µm.

Finally, we sought to investigate whether discrete biophysical subpopulations exist in human myofibroblasts. This is particularly important as force generation is a key aspect of the function of these cells. First, we quantified single-cell force profiles of human myofibroblasts (*n*=59 cells) during which hydrogel stiffness and imaging parameters were kept constant to facilitate comparison. Cell force profiles were normalised to cell area before input into principal component analysis (PCA) (Fig. 4A; Fig. S4A,B). Force profiles were created from meta-signatures of force measurements encompassing centiles of traction force and summary statistics for each cell. On inspection of the PCA plot, cells did not separate into obvious clusters (Fig. 4A; Fig. S4B). Instead, the PCA plot showed a small cluster of cells, with several more heterogeneous cell groups (Fig. 4A). PC1 captured the highest proportion of variance in the dataset (64.2%) and aligned cells based on their maximum and mean traction force without discrete clusters or subpopulations (Fig. S4A). Together, this suggests a continuum structure whereby myofibroblasts exist along a spectrum between high and low force-generating cells.

To identify potential clusters, we used unsupervised partitioning around medoids (PAM) clustering of the cells in principal component space with the parameter *k*=3 (Fig. 4D). Interestingly, each of the resulting clusters had distinct biophysical properties, with one composed of highly contractile cells. The second group exerted lower force, and there was a third intermediate population (Fig. 4D). Projecting the PAM clusters along the first principal component (PC) aligned each cluster with respect to maximum and mean traction force (Fig. 4E).

We then applied a separate computational workflow to resolve potential subpopulations. To partition single cells by their force profiles, we used an unsupervised clustering approach based on the Louvain algorithm, following recent approaches described for single-cell RNA-seq. For this, we plotted a *k*-nearest-neighbour (*k-*NN) graph for each pair of cells using the Euclidean distance between the scores of significant PCs to identify *k-*NNs (Fig. 4B,C). This graph was then refined to a shared nearest neighbour graph (SNN), informed by local neighbourhoods (Jaccard distance), and was then used as the input to the Louvain algorithm. Consistent with PCA projection, this method returned three biophysical clusters. Together, these data elucidate the presence of separate subpopulations of myofibroblasts with distinct biophysical properties, with a spectrum of high to low contractile cells existing simultaneously.

Finally, we integrated biophysical force measurements with morphological and cytoskeletal properties in myofibroblasts (Fig. 4F,G). For this, we performed immunofluorescence staining for F-actin on freshly isolated myofibroblasts on 4.5 KPa hydrogels, and at the same time point (60 min after seeding) we measured myofibroblast traction force. We quantified cell size, circularity, stress fibre length and orientation and used these metrics as an input for graph-based clustering. Remarkably, this again revealed three clusters with distinct cytoskeletal properties (Fig. 4F). One cluster was composed of large, polarized cells with highly organized and uniform stress fibres (‘high’ cluster). A second cluster was composed of intermediate size cells, with disorganized stress fibre length and orientation (‘int’ cluster). Finally, a third cluster was enriched for smaller cells exhibiting stress fibres with low orientation and length properties (‘low’ cluster) (Fig. 4F,G). Together, these show that topological cytoskeletal organization mirrors biophysical properties in myofibroblasts, with each composed of distinct subpopulations.

### Conclusions

In summary, we have characterised the biophysical profile of primary human myofibroblasts isolated from a fibrotic microenvironment in unprecedented detail. Employing a combination of unsupervised computational analysis and single-cell traction force microscopy as functional readout we resolved the complex spatiotemporal pattern of myofibroblast force generation. Building a fibrotic model through nano-indentation of native Dupuytren's nodules enabled the first measurements of myofibroblast force generation in a representative mechanical environment. Focal complexes have previously been described in myofibroblasts (Goffin et al., 2006), but our results extend beyond this to illustrate the evolution and mechanics of these structures on binding collagen. During cell spreading the magnitude of traction forces remained stable, but their distribution was highly dynamic. On initial contact with the collagen-coated matrix, myofibroblasts exert traction force at the cell periphery that transforms to form force foci. It could be reasoned that this phenomenon facilitates an initial phase of cell-ECM interaction promoting cell spreading, followed by a second phase that permits binding to and remodelling of matrix proteins.

We also uncovered distinct biophysical subpopulations of myofibroblasts. These discrete subpopulations were placed along a linear trajectory following both PCA and graph-based clustering, supporting the concept of these cells as existing along a continuum. This may reflect an underlying differentiation path or could represent a dynamic equilibrium within which cells flux between high and low contraction states.

Single-cell biology has uncovered many critical cellular processes, fostering a deep appreciation for the significance of heterogeneity in biology. To complement the quantification of biophysical profiles, we sought to integrate morphological and cytoskeletal parameters, and performed unsupervised clustering of myofibroblasts based on these properties. Although cell size and circularity did not directly influence traction forces in our experiment, we again noted three clear subpopulations. Indeed, cytoskeletal topology mirrored the biophysical profiles of myofibroblasts. Exploring the cytoskeletal clusters suggests that morphological heterogeneity was explained mostly by cell spreading and polarisation, with one large, highly polarised myofibroblast cluster, and two clusters of variable size with more disorganised actin organisation. This observation supports inferences made from our computational and experimental traction force analysis that suggest a unified continuum structure along which myofibroblasts transition between polarisation states. During this transition and cell spreading, large-scale modifications in traction force distribution and cytoskeletal structure were observed, but the magnitude of force generation remains stable. Moving forward, future work might also consider the time domain of this process.

Limitations of our study include the need for tissue digestion to isolate single cells, which likely exerts phenotypic effects on myofibroblasts. Also, although myofibroblasts are the predominant cell type in nodules, we performed no definitive enrichment step to isolate a pure myofibroblast population. With regards to the experimental protocol used, the necessity to obtain quantifiable bead displacements required the use of 4.5 kPa gels, which is the lower end of the force measurements in nodules. Looking forward, it would be important to integrate our single-cell biophysical and cytoskeletal profiling of myofibroblasts with other stromal cells to find potential conserved and distinct functional states. This would facilitate the development of detailed functional taxonomies of stromal cells in human disease. Our description of the biophysical readouts of primary human cells forms a foundation for future research that should aim to integrate functional parameters with gene expression and proteomic measurements to construct a validated cellular census of myofibroblasts.

## MATERIALS AND METHODS

### Patient samples

After approval by the local ethical review committee (REC 07/H0706/81), tissue samples were obtained with informed consent from patients with DD. Dupuytren's nodular tissue was obtained from individuals with DD undergoing dermofasciectomy.

### Cell culture

Cells from DD patients were isolated from α-SMA-rich nodules as described previously Verjee et al. (2009). Tissue samples were dissected into small pieces and digested in DMEM (Lonza) with Type I collagenase (Worthington Biochemical Corporation) +DNase I (Roche Diagnostics) for up to 2 h at 37°C. Cells were cultured in DMEM with 5% (vol/vol) FBS and 1% penicillin-streptomycin at 37°C in a humidified incubator with 5% (vol/vol) CO_2_. Cells before passage two were used for experiments.

### Immunofluorescence and confocal microscopy

Dupuytren's myofibroblasts were fixed with 4% paraformaldehyde in PBS for 20 min, longitudinally bisected, embedded in paraffin wax, and 7-μm sections from the cut surface were processed for immunofluorescence. The tissue sections were stained with Phalloidin-AF488 (Life Technologies). Nuclei were counterstained with DAPI (4, 6-diamidino-2-phenylindole; Sigma-Aldrich) and mounted using Prolong™ Gold anti-fade (Life Technologies). Fluorescent images were captured using a confocal system (Zeiss LSM 710).

### F-actin orientation analysis

The orientation properties of the actin filaments in myofibroblast immunofluorescence images (Phalloidin-488) were computed based on the evaluation of the structure tensor in a local neighbourhood using the Java plugin for ImageJ (http://imagej.nih.gov/) ‘OrientationJ’. After specifying the size of a Gaussian-shaped window, the program computes the structure tensor for each pixel in the image by sliding the Gaussian analysis window over the entire image. The local orientation properties are computed and are then visualised as gray-level or colour images with the orientation being typically encoded in colour. The data presentation was performed using the ‘ggplot2’ R package (R Version 3.5.). We analysed the F-actin orientations from at least 60 individual cells over the course of at least three independent experiments.

### Atomic force microscopy

Mechanical measurements were obtained using an Asylum Research MFP-3D atomic force microscope (Oxford Instruments). Fresh tissue samples from DD patients were dissected to obtain an approximately 10 µm cube from the centre of the nodule. This was then embedded in OCT and snap frozen in liquid nitrogen. Using a cryostat, 30 μm longitudinal slices were cut and mounted onto a glass slide. Tissue stiffness was measured through micro-indentation using a 0.072 Nm^−1^ probe with spherical 5 μm SiO_2_ tip (NovaScan) and the cantilever spring constant was calibrated using the thermal fluctuation method. 100 nano-indentation measurements were taken from 10×10 μm squares, with at least three squares per nodule, with the sample in double-distilled water at room temperature. Elastic modulus was calculated using the Hertz model from indentation profiles using MFP-3D software (Oxford Instruments and Igor).

### Preparation of polyacrylamide hydrogels

Polyacrylamide (PAA) gels were prepared as previously described in Colin-York et al. (2017). Briefly, 4.5 kPa polyacrylamide gels were prepared by combining acrylamide monomers (Sigma-Aldrich) at 10% and bis-acrylamide cross-linkers (Sigma-Aldrich). Polymerization was initiated by the addition of TEMED (Sigma-Aldrich) followed by 10% Ammonium persulfate (Sigma-Aldrich) at a volume ratio of 1:250 and 1:100, respectively. The gel solution was pipetted between two glass coverslips, one of which had been treated with APTMS 0.5% (Sigma-Aldrich) followed by 0.5% glutaraldehyde (Sigma-Aldrich) to firmly attach the gel to the coverslip.

PAA functionalization was achieved using the ultraviolet (UV) activated cross-linker Sulfo-SANPAH (Thermo Fisher Scientific). Each gel was coated with 20 mg per ml solution of Sulfo-SANPAH and exposed to 365 nm UV light for 10 min. The gel was then washed to remove any excess cross-linker and then coated with a 100 µg/ml Type I Collagen (First Link) and incubated at 37°C for 1 h. Gels were then washed and incubated at 37°C before cell seeding.

### Traction force microscopy

Live cell imaging was performed at 37°C in Phenol-Red-free DMEM (Gibco 2106309) without serum. Images were acquired using an inverted wide-field confocal microscope (Zeiss LSM 710) fitted with a stage incubator (5% CO^2^ in air, 37°C). Images of fluorescent beads (fiducial markers) were acquired in the Texas Red channel (580/605 nm) and cells acquired in the Alexa-Fluor 488 channel (488 nm). Imaging was performed using a 63× (1.4 NA) oil objective and images were processed using ImageJ software. Average pixel size was 153.3 nm. Cells were removed from the gel surface using Tryspin (0.5%) after 60 min.

Data processing was performed as described in Colin-York et al. (2017) using ImageJ plugins. First, images were imported into ImageJ using Bioformats and corrected for experimental drift between each image using a template-matching and slice-alignment plugin. A normalised correlation-coefficient-matching method was implemented with subpixel registration. Following this, particle image velocimetry (PIV) was applied to quantify bead displacements using a cross-correlation algorithm with an iterative window size of 64 pixels and 32 pixels yielding displacement vectors of 16 pixels and a final resolution of 2.4 μm. Post-processing of PIV vectors was undertaken using a normalised median test (noise level of 0.2 and threshold of 5.0) to filter and replace erroneous displacement vectors with the median value from nearby vectors (*n*=30). This process compares each vector with its 30 nearest neighbours and corrects for inaccurate displacement vectors that result from noise in the raw images.

Fourier transform traction cytometry (FTTC) was used to reconstruct traction forces from displacements fields. The parameters used in the FTTC code included a pixel size of 0.153 μm, Poisson's ratio of 0.5 and the elastic modulus of the gel was 4.5 kPa. A gel stiffness of 4.5 KPa was used to obtain quantifiable bead displacements. The appropriate regularisation factor λ was determined empirically as per Schiller et al., 2013. This value was selected to minimise the contribution of noise and optimise traction force recovery from raw image sequences. A value of 1e^−10^ was selected and kept constant throughout all experiments.

### Computational analysis

#### Feature selection and dimensional reduction

Downstream analysis and visualisation were performed using MATLAB (Mathworks) and R (R Version 3.5). We first constructed meta-signatures for single-cell force profiles using percentiles and summary statistics (mean, max, median and standard error) and normalised these to cell area. Next, normalised force profiles were centred and scaled before input to principal component analysis, implemented using the ‘prcomp’ function from the ‘stats’ R package. To initially partition cells, we used unsupervised PAM clustering of the cells in principal component space with the parameter *k*=3.

#### *k*-NN-graph-based clustering

After PCA, significant principal components were identified using the permutation test implemented using the ‘permutationPA’ function from the ‘jackstraw’ R package. This test identified seven significant PCs and these were used as input for graph-based clustering. To cluster single cells by their force profiles, we used unsupervised clustering based on the Louvain community-detection algorithm. For this, we first constructed a *k-*NN graph using, for each pair of cells, the Euclidean distance between the scores of significant principal components as the metric. The *k-*NN graph was computed using the function ‘nng’ from the R package ‘cccd’. After this, the *k*-NN graph was refined using the shared local neighbourhoods of points (Jaccard Distance) and clustered using the ‘louvain_cluster’ function from the ‘igraph’ R package.

## Supplementary Material

Supplementary information
