# Supplementary material for: Single cell force profiling of human myofibroblasts reveals a biophysical spectrum of cell states
Source: Biol Open. 2020 Mar 24;9(3):bio049809. doi: 10.1242/bio.049809 (PMC7104857; doi:10.1242/bio.049809)
Supplement: Supplementary information [file biolopen-9-049809-s1.pdf]

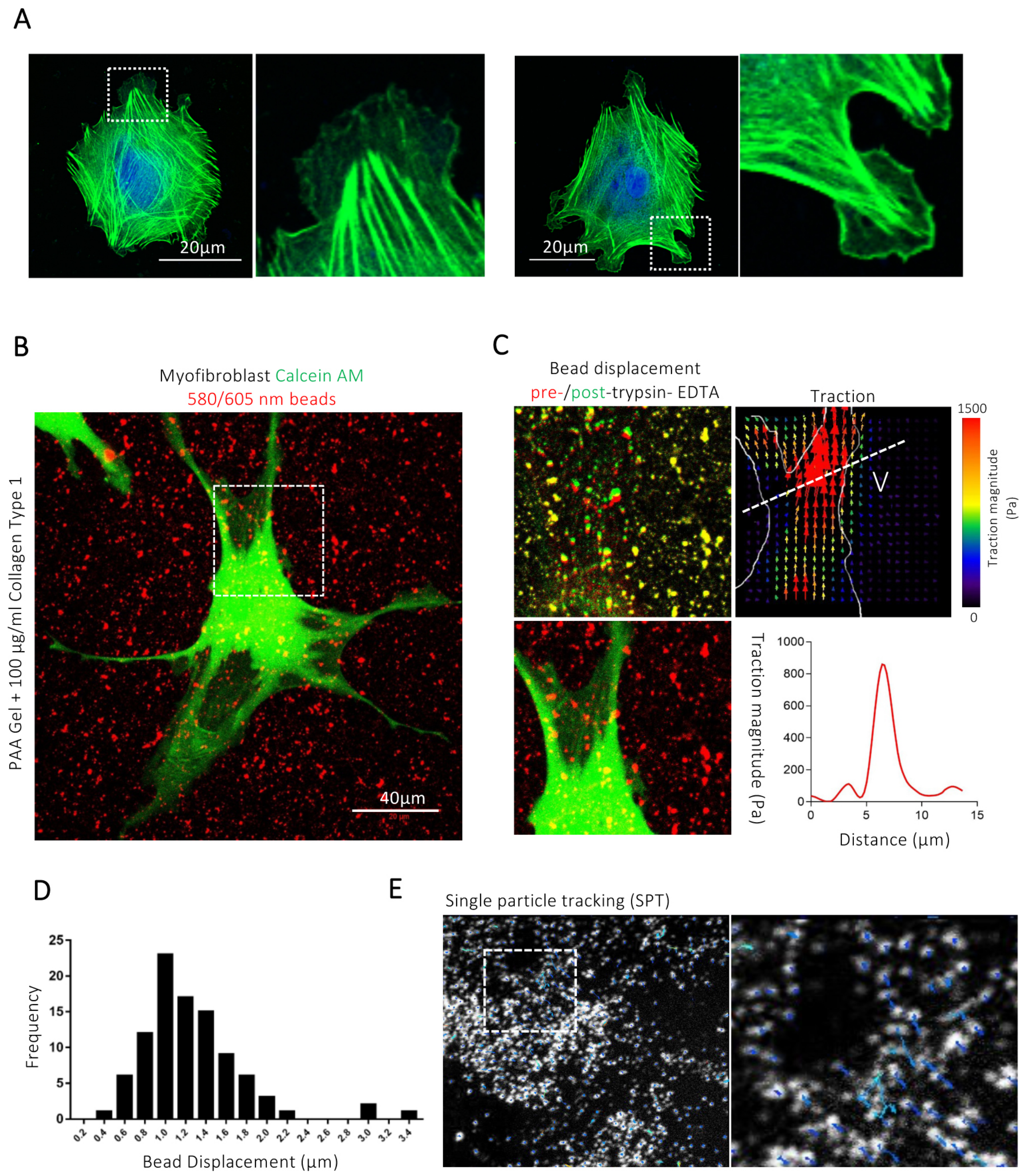

Fig S1. Experimental and computational setup for high resolution TFM
A) Confocal images of DD myofibroblasts on the surface of 4.5kPa hydrogel stained with Phalloidin-AF488 (Green) labelling F-actin. Cells were seeded on hydrogels 60 minutes prior to staining to confirm stress fibre formation on softer environments. Right panels are zoomed regions highlighted by white dashed squares. B) Confocal image of myofibroblast tagged with Calcein AM (green) on 4.5kPa PAA gel with fluorescent beads (red) on the surface. C) Image analysis workflow of traction force microscopy (TFM). Top left, confocal image from white square in (B) demonstrating bead displacement on gel surface before and after addition of trypsin. Top right, corresponding force vector plot. Bottom left, confocal image of myofibroblasts from white dashed square in Figure 1B. Bottom right, line profile of Line V in (B) showing linear profile of peak traction force generation from one human myofibroblast. D) Histogram of bead displacements from > 50 cells from three independent experiments with cells seeded on to 4.5KPa hydrogels. E) Single particle tracking (SPT) of fiducial markers on PAA gel surface. Magnification x63. White points represent beads and tracks signify movement after removing cell from gel surface.

Biology Open • Supplementary information

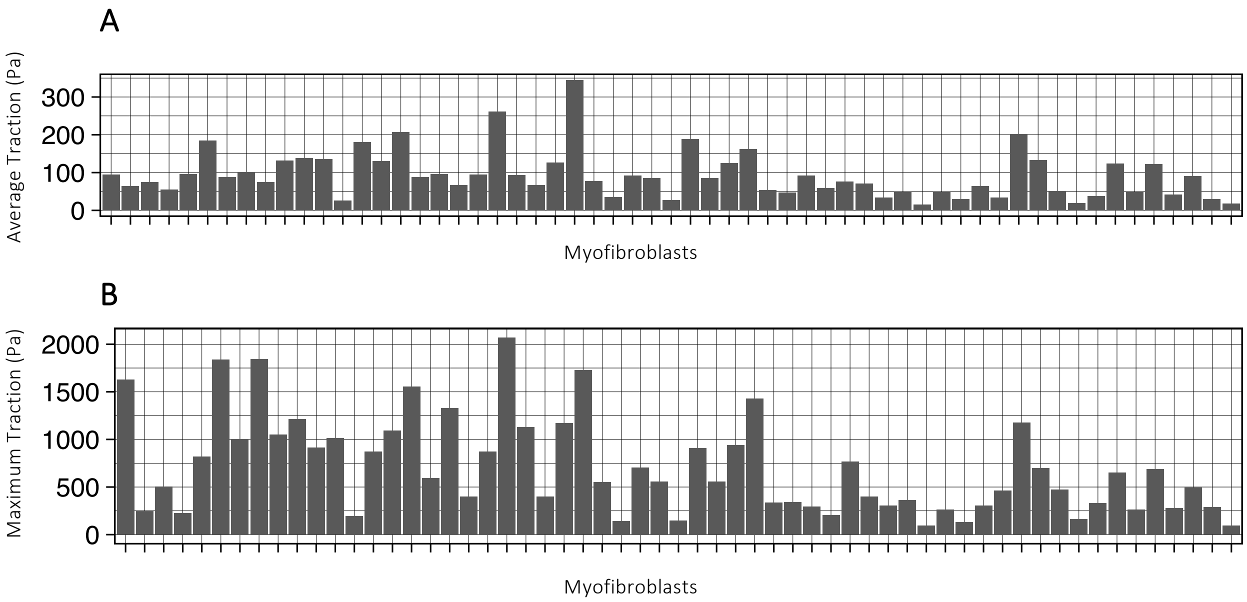

Fig S2. Cellular traction forces of human myofibroblasts.
A-B) Bar plots demonstrating average normalized (A) and peak (B) traction force in myofibroblasts on 4.5kPa PAA hydrogels (*n* = 46 cells from >10 independent donors). X axis represents individual myofibroblasts. Pa = Pascal.

Biology Open • Supplementary information

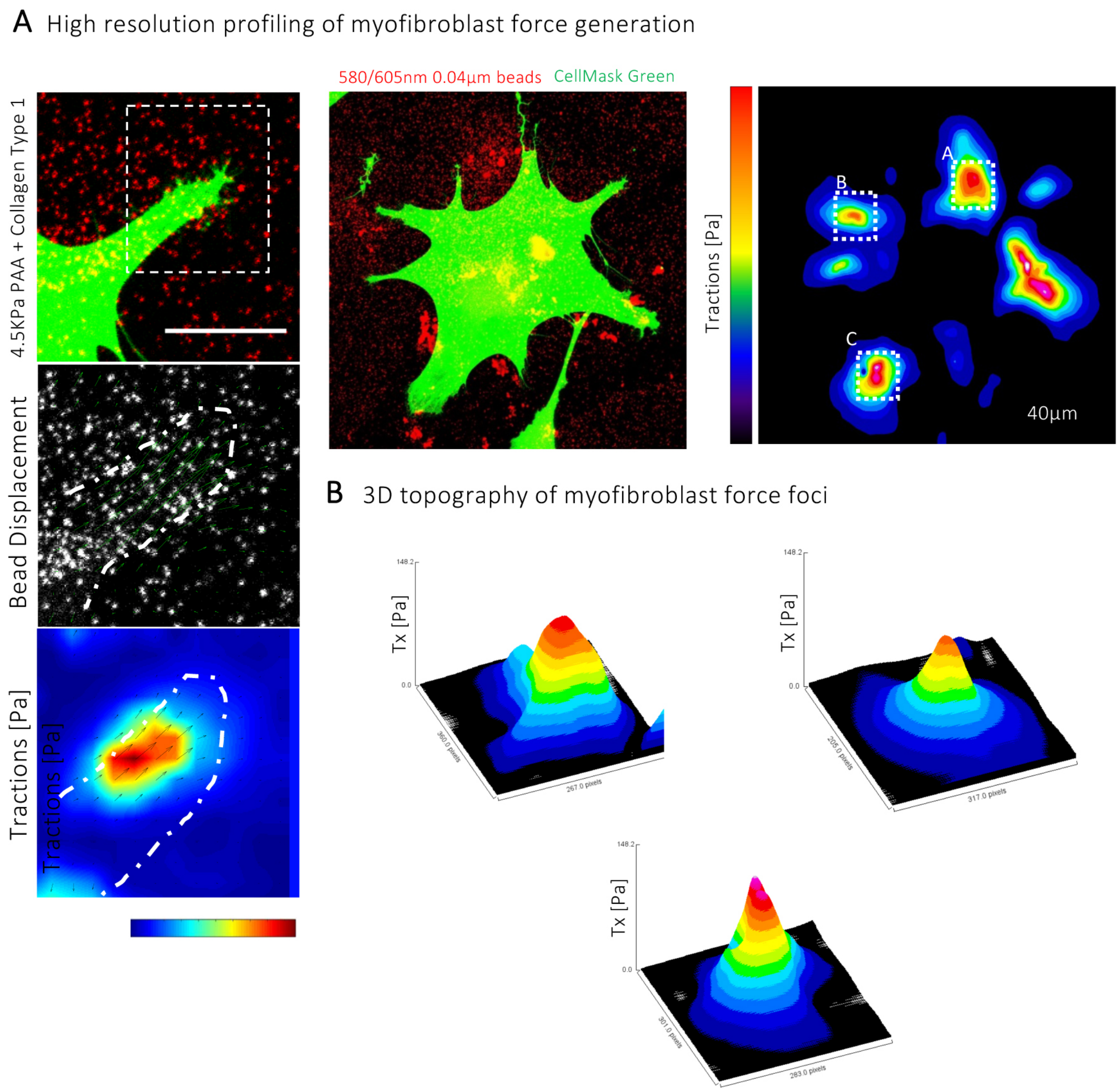

Fig S3. Myofibroblast traction forces are discrete and localized.
A) Traction stress heatmap and corresponding confocal image of Calcein AM tagged myofibroblast (Green) on 4.5 kPa PAA gel with marker beads (Red) showing localized area of high traction force in red. B) 3D surface plots of regions indicated by three white lines in (A).

Biology Open • Supplementary information

A

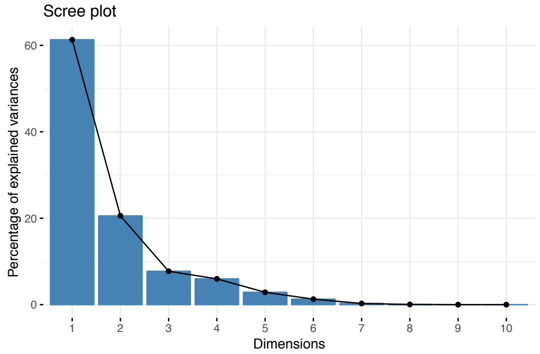

B

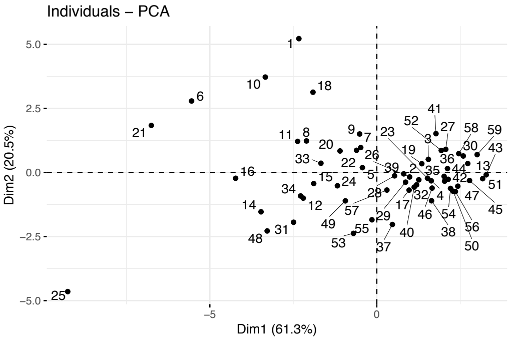

C

0	Quantile 1	Quantile 2	Quantile 2	Quantile 4	Mean	Max	Min	Median
-73.949	16.511	37.438	88.196	1627.742	94.08010284	1627.742	-73.949	37.438
0.188	35.3585	50.74	79.088	246.718	64.4955709	246.718	0.188	50.74
-8.78	14.676	29.175	100.238	501.704	74.87926362	501.704	-8.78	29.175
3.404	27.246	46.2035	77.001	224.089	55.49664636	224.089	3.404	46.2035
-3.998	34.647	62.007	117.774	817.476	95.58946676	817.476	-3.998	62.007
-70.163	62.56275	115.0595	220.842	1838.217	184.8466745	1838.217	-70.163	115.0595
-12.92	26.022	51.427	108.9345	1003.883	88.03194957	1003.883	-12.92	51.427
-13.822	33.569	63.29	123.352	1845.207	100.9565914	1845.207	-13.822	63.29
-21.027	26.431	48.174	91.267	1048.177	74.42248851	1048.177	-21.027	48.174
-71.897	39.31675	80.8835	171.86925	1211.963	131.1515944	1211.963	-71.897	80.8835
-37.907	35.822	92.1645	197.32625	913.714	137.7150864	913.714	-37.907	92.1645
-2.581	56.502	110.495	165.681	1013.564	135.6739786	1013.564	-2.581	110.495
0.423	10.2305	18.068	33.475	196.014	26.162897	196.014	0.423	18.068
-11.113	81.105	135.235	219.982	871.862	180.8450832	871.862	-11.113	135.235
-6.613	52.7185	85.0335	167.493	1090.56	130.3096729	1090.56	-6.613	85.0335
-21.131	46.7495	125.356	256.2575	1554.105	207.2889641	1554.105	-21.131	125.356
-0.247	30.676	52.118	101.316	593.399	87.35433952	593.399	-0.247	52.118
-54.857	25.828	55.294	105.39025	1329.1	96.29688204	1329.1	-54.857	55.294
-9.882	17.405	36.98	89.893	401.344	66.24065976	401.344	-9.882	36.98
-16.115	43.821	71.351	116.669	872.241	94.35488568	872.241	-16.115	71.351
-65.143	54.98	125.433	356.497	2067.478	260.7961501	2067.478	-65.143	125.433
-14.379	24.362	47.1765	119.475	1130.176	93.59135519	1130.176	-14.379	47.1765
-1.643	19.885	36.863	90.003	396.879	66.38333143	396.879	-1.643	36.863
1.344	32.795	61.019	197.8635	1172.052	125.6989447	1172.052	1.344	61.019
-5.879	131.0705	260.259	459.09	1727.203	344.8636953	1727.203	-5.879	260.259
-11.997	19.38525	55.2185	103.543	549.461	77.8732799	549.461	-11.997	55.2185
-20.615	15.808	25.706	49.534	143.72	34.47582742	143.72	-20.615	25.706
-14.078	30.709	49.311	112.414	704.702	91.48031991	704.702	-14.078	49.311
-10.77	22.663	52.672	125.7995	557.303	85.91640882	557.303	-10.77	52.672
-0.45	12.375	21.66	35.71025	147.945	26.96299115	147.945	-0.45	21.66
1.834	25.219	129.842	281.052	908.756	188.5235953	908.756	1.834	129.842
-6.426	19.811	42.63	124.263	555.155	84.6542758	555.155	-6.426	42.63
-25.391	42.233	77.002	161.474	942.072	124.9798771	942.072	-25.391	77.002
-0.421	38.8225	80.1485	227.60475	1430.395	162.1959635	1430.395	-0.421	80.1485
-3.051	15.357	28.4555	63.279	333.604	53.32546786	333.604	-3.051	28.4555
-6.804	13.9835	24.2	48.9035	340.872	46.47724549	340.872	-6.804	24.2
7.033	51.23225	77.628	121.3845	294.6	92.41150286	294.6	7.033	77.628
2.801	27.389	52.227	84.321	204.228	59.25361872	204.228	2.801	52.227
-0.879	21.554	35.304	71.018	764.737	75.67327553	764.737	-0.879	35.304
-5.947	24.85375	50.338	98.65825	401.037	71.38030113	401.037	-5.947	50.338
-18.287	16.484	27.084	40.971	303.583	33.31712864	303.583	-18.287	27.084
-1.6	15.873	29.91	62.88	363.333	48.56106402	363.333	-1.6	29.91
0.309	6.99375	10.747	18.508	96.291	15.13409987	96.291	0.309	10.747
-4.478	15.3015	28.04	66.8585	264.586	47.69786395	264.586	-4.478	28.04
-1.205	12.85525	21.0225	37.96425	129.336	30.13667467	129.336	-1.205	21.0225
-3.9	20.087	42.172	76.75	306.733	64.62686512	306.733	-3.9	42.172
-1.227	15.495	27.5505	46.37775	463.838	33.64296456	463.838	-1.227	27.5505
-0.446	46.493	129.329	289.816	1174.672	201.4557806	1174.672	-0.446	129.329
-20.873	45.772	91.688	170.936	696.612	133.0535466	696.612	-20.873	91.688
0.007	14.54875	28.3315	60.94325	475.047	51.56099115	475.047	0.007	28.3315
0.444	7.4195	11.571	19.4375	164.98	19.19321317	164.98	0.444	11.571
-6.605	16.951	28.334	43.607	331.359	37.88496623	331.359	-6.605	28.334
-2.253	51.834	103.293	174.0505	652.082	124.1927785	652.082	-2.253	103.293
-1.93	16.854	28.7775	63.69675	264.938	48.39036521	264.938	-1.93	28.7775
-4.02	40.277	77.922	166.701	689.094	122.8483949	689.094	-4.02	77.922
0.769	17.88075	28.4095	47.81075	279.397	41.52593367	279.397	0.769	28.4095
-3.381	39.90125	76.7955	123.13925	491.334	90.85562532	491.334	-3.381	76.7955
-1.128	11.383	18.688	32.672	288.166	30.33645792	288.166	-1.128	18.688
-0.005	7.611	13.657	23.119	94.021	18.04378415	94.021	-0.005	13.657

Biology Open • Supplementary information

Fig S4. Principal component analysis of human myofibroblast force signatures.
A) Bar plot with overlay of line plot (Scree plot) showing variance explained by each principal component in single cell force profiles. Dimensions is principal components. B) Scatter plot projecting single cell force profiles along the first two principal components (Dim1 and Dim2). Each point represents one cell, and each is labelled by the order in which it was collected (*n* = 59, from >5 independent donors). C) Summary statistics for clustering.

Biology Open • Supplementary information
